# Age- and Microbiota-Dependent Cell Stemness Plasticity Revealed by Cattle Cell Landscape

**DOI:** 10.34133/research.0025

**Published:** 2023-01-13

**Authors:** Jia-Jin Wu, Senlin Zhu, Yi-Fan Tang, Fengfei Gu, Teresa G. Valencak, Jian-Xin Liu, Hui-Zeng Sun

**Affiliations:** ^1^ Institute of Dairy Science, College of Animal Sciences, Zhejiang University, Hangzhou 310058, China.; ^2^ Ministry of Education Key laboratory of Molecular Animal Nutrition, Zhejiang University, Hangzhou 310058, China.; ^3^ Ministry of Education Innovation Team of Development and Function of Animal Digestive System, Zhejiang University, Hangzhou 310058, China.

## Abstract

Newborn ruminants are considered functionally monogastric animals. The poor understanding of cellular differences between newborn and mature ruminants prevents the improvement of health and performance of domestic ruminants. Here, we performed the single-cell RNA sequencing on the rumen, reticulum, omasum, abomasum, duodenum, jejunum, ileum, cecum, colon, rectum, liver, salivary gland, and mammary gland from newborn and adult cattle. A comprehensive single-cell transcriptomic atlas covering 235,941 high-quality single cells and 78 cell types was deciphered. A Cattle Cell Landscape database (http://cattlecelllandscape.zju.edu.cn) was established to elaborately display the data and facilitate effective annotation of cattle cell types and subtypes for the broad research community. By measuring stemness states of epithelial cells in each tissue type, we revealed that the epithelial cells from newborn forestomach (rumen, reticulum, and omasum) were more transcriptionally indistinct and stochastic compared with the adult stage, which was in contrast to those of abomasum and intestinal tissues. The rapid forestomach development during the early life of calves was driven by epithelial progenitor-like cells with high DNA repair activities and methylation. Moreover, in the forestomach tissues of newborn calves, the *Megasphaera* genus was involved in regulating the transcriptional plasticity of the epithelial progenitor-like cells by DNA methylation regulation. A novel cell type, the *STOML3^+^* cell, was found to be newborn-specific. It apparently plays a crucial role in stemness maintenance of its own and cholangiocytes in the hepatic microenvironment. Our results reveal that the age- and microbiota-dependent cell stemness plasticity drives the postnatal functional maturity of ruminants.

## Introduction

During the Neolithic, ruminants were domesticated to provide meat and milk for humans [1]. They have highly organized digestive systems including forestomach (rumen, reticulum, and omasum), abomasum, small intestines, large intestines, and accessory glands [2,3]. Nutrients absorbed by the gastrointestinal tract (GIT) are delivered to the liver for catalytic metabolic processes to enable cell growth and production. The newborn (NB) ruminant GIT is functionally equivalent to that of monogastric animals and displays marked changes in morphology, structure, and function before reaching full ruminating capacity [4]. For example, the rumen expands gradually after birth to more than 70% of the GIT volume [5,6]. The maturation of the ruminating system starts after birth and is orchestrated by diverse cell types across multiple tissues. During this process, the digestion undergoes a shift from primarily intestinally absorbed colostrum- and milk-derived nutrients to short-chain fatty acids, amino acids, and other compounds from feed and ruminal microbial sources; the metabolic function of the liver changes from being primarily responsible for ketogenesis and glycolysis to gluconeogenesis using propionate as a substrate [4].

The molecular mechanisms behind the postnatal ruminating maturation is considered as ontogenic [7], with the mechanisms yet not being completely understood. However, there are recent efforts to link the host transcriptome with microbiota using lamb or calf models [7,8]. The bovine GIT tissues undergo rapid cell proliferation and differentiation in the postnatal period [9], and organogenesis is dependent on cell stemness of individual cells [10,11]. There is little information available on the unique cellular differentiation markers of bovine cell lineages [12,13], which hinders the determination of stemness states of individual cells and prevents a full understanding of the molecular mechanisms relating to postnatal maturation of the ruminating system. The advances on single-cell RNA sequencing (scRNA-seq) allow the disentangling of heterogeneity in cell composition and cell-type-specific dynamic changes in time of organ development [14,15]. Novel algorithms for quantitatively measuring cellular stemness states at the single-cell level from scRNA-seq data have emerged [11] and were successfully applied in a recent study on human organ development by assessing single-cell stemness [16]. In our previous study, we provided novel insights into cell composition and potential cell functions at single-cell resolution in adult (AD) dairy cattle [17]. However, alterations in cell type composition and stemness at multiple temporal and spatial scales have not been investigated in sufficient detail yet.

The postnatal functional maturity of ruminants is mainly due to the changes in the composition and function of various tissue cell types and microbiota in the digestive system. To investigate the biological mechanisms of postnatal functional maturity in ruminants, we firstly used scRNA-seq to build a comprehensive single-cell compendium across 13 bovine tissue types from the NB and the AD stages and delineated the multitissue patterns of cell stemness plasticity. Next, we integrated the epithelial microbiome, metabolome, and single-cell transcriptome to investigate the role of microbiota in the cell stemness plasticity of the forestomach. Finally, the new cross-species comparison based on single-cell transcriptomic data will extend our knowledge on cattle-specific novel cell types contributing to the maintenance of cell stemness in postnatal maturation of the ruminating system.

## Results

### Construction of a cattle cell atlas

**Fig. 1.**
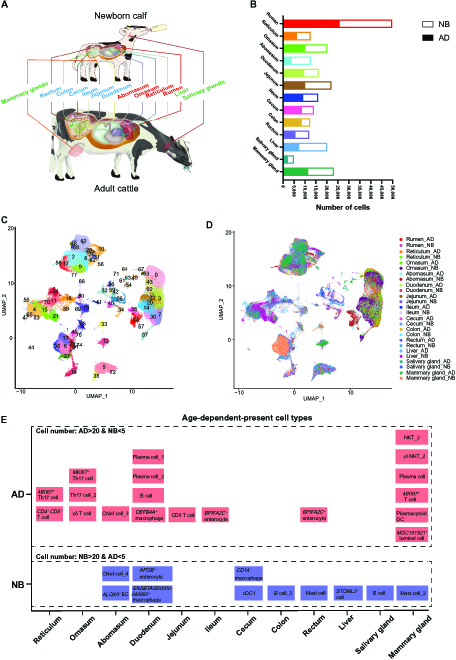
Construction of single-cell compendium of 13 tissue types of the newborn (NB) and adult (AD) cattle. (A) Thirteen tissue types are used for scRNA-seq in NB and AD cattle. (B) The bar plot shows the number of high-quality single cells per tissue type in NB and AD cattle. (C and D) Uniform manifold approximation and projection (UMAP) plot of all cells, colored by cell clusters (C) and tissues (D); *n* = 235,941 single cells. (E) Representative age-dependent-present cell types. Only the cell types with more than 20 cells in AD cattle and less than 5 cells in NB cattle are considered as AD-dependent-present cell types. Cell types with more than 20 cells in NB cattle and less than 5 cells in AD cattle are considered as NB-dependent-present cell types. DC, dendritic cell; cDC1, conventional type 1 dendritic cell; NKT, natural killer T; EC, epithelial cell.

To delineate the age-dependent cell types and transcriptional profiles of various tissues of highly organized digestive systems in cattle, we used 10x Genomics based scRNA-seq to establish a profile of forestomach (rumen, reticulum, and omasum), abomasum, small intestines (duodenum, jejunum, and ileum), large intestines (cecum, colon, and rectum), liver, salivary gland, and mammary gland tissues of both NB and AD cattle (Fig. 1A and Tables S1 and S2). After removing low-quality single cells and doublets (see Materials and Methods), 235,941 high-quality cells were retained: 49,689 cells from rumen, 12,844 from reticulum, 20,195 from omasum, 12,637 from abomasum, 16,222 from duodenum, 22,027 from jejunum, 16,001 from ileum, 13,987 from cecum, 12,383 from colon, 11,848 from rectum, 20,066 from liver, 4,894 from salivary gland, and 23,148 from mammary gland (Fig. 1B).

We combined all the 235,941 cells into the cell clustering analysis across tissues with batch effect correction (see Materials and Methods) and identified 78 cell clusters (Fig. 1C). The annotation of cell clusters was performed by both data-driven analysis and manual interpretation based on the highly expressed marker genes. Detailed information on the descriptions of cell types and their highly expressed marker genes are available in Table S3 and Fig. S1. A uniform manifold approximation and projection (UMAP) of the single-cell dataset (Fig. 1D) revealed that cells were organized primarily by tissue type rather than age. Therefore, to further systematically investigate the dynamics of cell type composition between NB and AD, we performed cell clustering analysis of each tissue type separately (see Materials and Methods). We next explored the cell types that were age-dependent-present in each tissue type. In a single tissue type, any cell type with more than 20 cells in AD cattle and less than 5 cells in NB cattle was considered as AD-dependent-present cell type and otherwise was considered as NB-dependent-present cell type. By comparing the numbers of each epithelial or immune cell types between NB and AD stages in each tissue, we discovered the age-dependent-present cell types (Fig. 1E). For example, *MKI67^+^* Th17 cells were enriched in the AD reticulum and omasum tissues; the chief cell_3 was enriched in the AD abomasum, whereas the *ALOX5^+^* epithelial cell and the chief cell_4 were enriched in the NB abomasum; the *BPIFA2C^+^* enterocyte was enriched in the AD rectum, whereas the mast cell was enriched in the NB rectum.

**Fig. 2.**
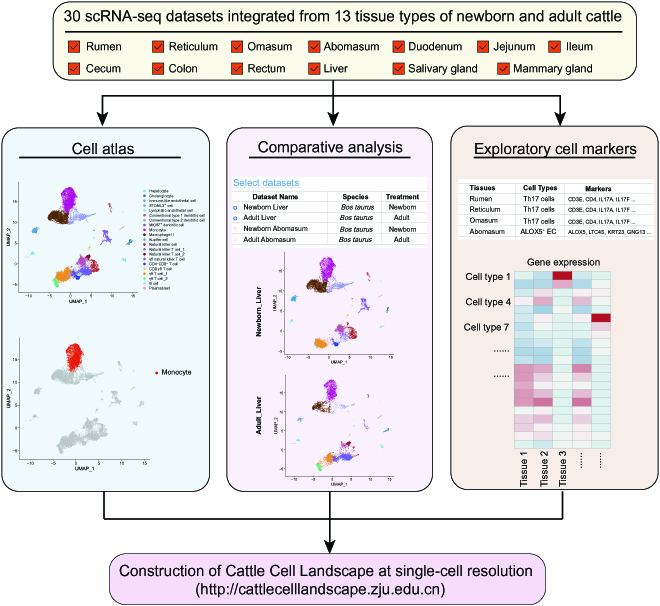
The schematic of the scRNA-seq datasets and functional modules that are included in the CCL website.

Furthermore, we constructed the Cattle Cell Landscape (CCL) database (Fig. 2; http://cattlecelllandscape.zju.edu.cn), a comprehensive web resource consisting of 30 single-cell datasets across 13 tissue types. The results of cell clustering, cell-type annotation, and marker gene identification in single or multiple single-cell datasets can be available for public access and interactive exploration by using the user-friendly interface in the CCL. The CCL offers users 2 modules, the “Landscape Datasets” module and the “Marker Genes Search” module, to explore the single-cell datasets. In addition to the visualization of the single tissue-type dataset, a comparative analysis of cell types between NB and AD groups is also offered in the CCL. Our scRNA-seq analysis revealed special marker genes for distinguishment of different cell types and thus presents an accurate and comprehensive resource of marker genes for cell types across various cattle tissues.

### Multitissue representation of cell stemness plasticity paradigm

**Fig. 3.**
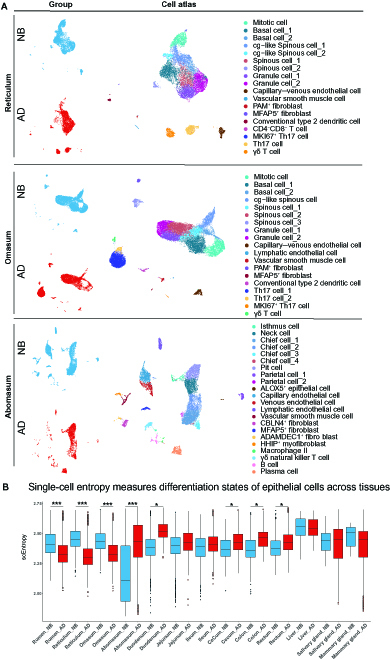
Dynamics of cell composition and cell stemness in tissue types between newborn (NB) and adult (AD) cattle. (A) Cell atlases of all cell types in each tissue among the 2 groups (NB, blue; AD, red). (B) Single-cell entropy of epithelial cells in each NB and AD tissue pair. *P* value < 0.0001 as the threshold for significance. * and *** represent *P* < 0.0001, *P* < 1.0 × 10^−100^, and *P* < 1.0 × 10^−200^, respectively.

Previous studies reported that the physiological changes between NB and AD cattle were associated with the GIT epithelium [18,19], and the organogenesis is dependent on cell stemness of individual cells [10,11]. However, little is known about how epithelial cell populations and stemness status of GIT and other key metabolic tissues are changed between the NB and AD stages. Therefore, we focused on the epithelial cellular composition of each organ separately (Fig. 3A to C and Figs. S2 to S5). Firstly, we compared the epithelial cell differentiation states between NB and AD cattle by calculating the single-cell entropy using the SLICE algorithm [11]. The results showed that there was no significant difference in the entropy of epithelial cells of the jejunum, ileum, liver, salivary gland, and mammary gland tissues between the 2 groups (Fig. 3D). Interestingly, when compared with the AD group, NB epithelial cells from the abomasum and some gut tissues (duodenum, cecum, colon, and rectum) showed significantly lower entropy (*P* < 0.0001), while the forestomach (rumen, reticulum, and omasum) epithelial cells had significantly higher (*P* < 1.0 × 10^−200^) entropy in the NB groups (Fig. 3D). Higher entropy is associated with larger cell stemness and functional uncertainty [11]. These results suggest that the high differentiation potential of forestomach epithelial cells at NB stage is likely the origin of the drastic changes in organ development and functional transition from preruminating to ruminating.

**Fig. 4.**
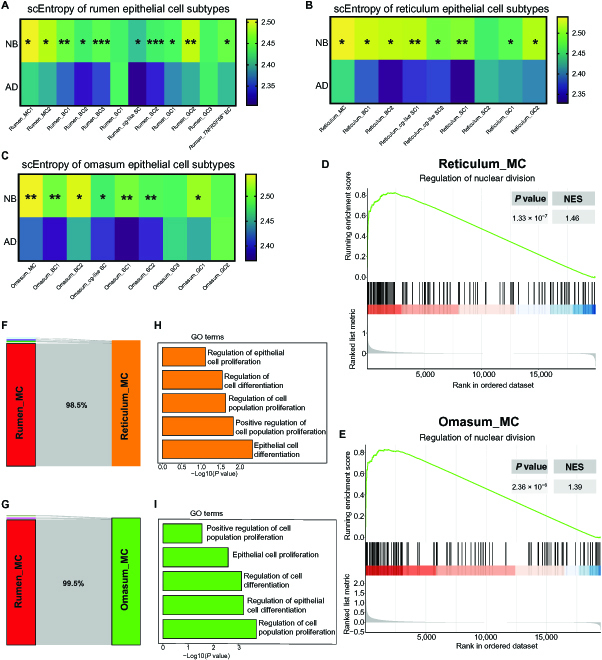
Dynamics of cell stemness in the epithelial cell subtypes of forestomach tissues between newborn (NB) and adult (AD) cattle. (A to C) Heatmap showing the single-cell entropy of epithelial cell subtypes in the NB and AD rumen (A), reticulum (B), and omasum (C) tissue pair. *P* value < 0.0001 as the threshold for significance. *, **, and *** represent *P* < 0.0001, *P* < 1.0 × 10^−100^, and *P* < 1.0 × 10^−200^, respectively. (D and E) GSEA identified the biological processes enriched in the mitotic cells (MCs) of the reticulum (D) and omasum (E) tissues. (F and G) The random forest classification of MC from reticulum (F) and omasum (G) tissues using MC from rumen samples as a training class. (H and I) Representative upregulated Gene Ontology (GO) terms in the MC of NB reticulum (H) and NB omasum (J). BC, basal cell; GC, granule cell; SC, spinous cell.

The forestomach tissues undergo rapid growth and development after birth. A previous study found that the most significant transcriptional differences between NB and AD ruminants were observed in the forestomach tissues by exploring transcriptomes of the entire GIT tissues during the transition from preruminant to ruminant [19]. However, which cell types drive the differences in transcriptional plasticity between NB and AD forestomach tissues remains unknown. Therefore, we next examined forestomach tissues to further explore cell stemness at the epithelial cell-subtype level. By comparing the single-cell entropy of the epithelial cell subtypes between NB and AD groups in the rumen (Fig. 4A), reticulum (Fig. 4B), and omasum (Fig. 4C), we found that almost all the epithelial cell subtypes in the NB group had significantly higher entropy than in the AD group in these 3 tissues. Remarkably, the mitotic cells (MCs; highly expressing *MKI67* and *KRT14*) had the highest entropy among all the epithelial cell subtypes in these 3 tissues. The gene set enrichment analysis (GSEA) also showed that MC had a high potential for cell division (Fig. 4D and E). The random forest classification revealed that the MC in the reticulum and omasum had similar identities (98.5% and 99.5%, respectively) to those in the rumen (Fig. 4F and G). By assessing the similarities of epithelial cell subtypes in the forestomach tissues using the MetaNeighbour analysis [20], we also observed that the gene expression patterns of MC clusters were conserved among the rumen, reticulum, and omasum at both NB and AD stages (area under the receiver operating characteristics [AUROC] score ≥ 0.95; Fig. S6). In addition to MC clusters, only 2 and 3 cell type clusters were conserved across the rumen, reticulum, and omasum at the NB and AD stages, respectively (AUROC score ≥ 0.95; Fig. S6). In other words, the similarities of most epithelial cell subtypes among the rumen, reticulum, and omasum were not conserved at both NB and AD stages (AUROC score < 0.95; Fig. S6) although the forestomach tissues are composed of the similar stratified squamous epithelium.

**Fig. 5.**
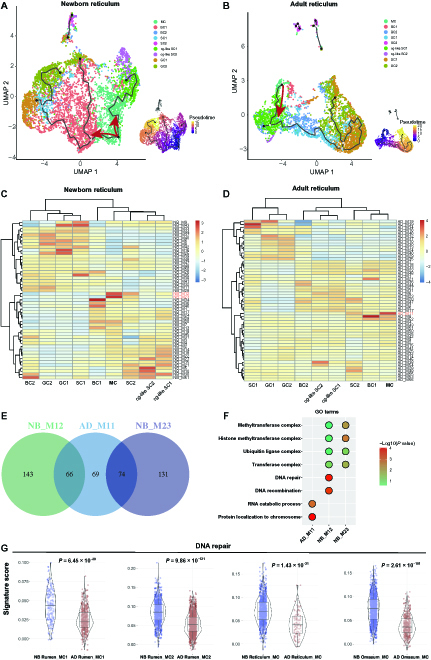
Differences of the MC differentiation patterns between newborn (NB) and adult (AD) forestomach tissues. (A and B) Pseudotime trajectory analysis corresponds to the differentiation of epithelial cells in the NB reticulum (A) and AD reticulum (B). (C and D) Gene modules that change as cells progress along the trajectory of NB reticulum (C) and AD reticulum (D). The colors from blue to red indicate low to high aggregate module scores. (E) Venn diagram shows unique genes of the AD_ M11, NB_M12, and NB_M23 when compared with each other. (F) Representative upregulated GO terms of unique genes in the AD_ M11, NB_M12, and NB_M23. (G) Gene scoring analysis for MCs in the NB and AD groups of forestomach tissues using the “DNA repair” gene set. BC, basal cell; GC, granule cell; SC, spinous cell.

Herein, to further unveil the molecular events associated with differentiation states within the MC, the differentially expressed genes (DEGs) were identified in NB and AD cattle. The upregulated DEGs of the MCs in the reticulum and omasum were enriched in the epithelial cell differentiation and cell proliferation, which is consistent with our previous findings in the rumen tissue [21]. Thus, the MC in forestomach tissue may possess different differentiation patterns in the early postnatal period compared with adulthood. To confirm this, we then performed pseudotime analysis of the reticulum epithelial cell subtypes and found that the MC of both NB and AD were located at the starting point of the differentiation trajectories (Fig. 5A and B), suggesting that MC could maintain continuous regeneration of stratified epithelium. Moreover, 2 bifurcations were displayed in the NB MC (Fig. 5A) but only 1 in the AD MC (Fig. 5B), indicating that the former MC might have more diverse differentiation patterns.

Finally, we explored modules of coregulated genes that were differentially expressed in the different paths through the trajectories. We identified 51 (NB_M1-M51) and 56 gene modules (AD_M1-M56) in the differentiation trajectories of NB and AD groups, respectively (Fig. 5C and D). The NB_M12 and NB_M23 were enriched in the NB MC (Fig. 5C), and the AD_M11 was enriched in the AD MC (Fig. 5D). Interestingly, compared with each other, AD_M11, NB_M12, and NB_M23 had many unique genes (Fig. 5E). The unique genes of NB_M12 and NB_M23 were enriched in DNA repair and methyltransferase complex, respectively (Fig. 5F). Gene set score analysis also showed that the MC in the NB group had high DNA repair (Fig. 5G). The higher activities of DNA repair and epigenetic modification may help to explain the high transcriptional plasticity observed in NB MC.

### Microbiota-dependent transcriptional plasticity in the forestomach tissues of NB cattle

**Fig. 6.**
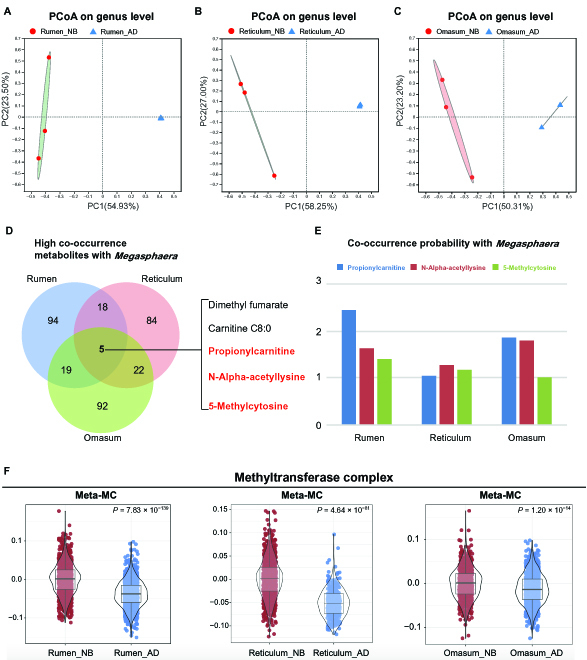
*Megasphaera*-associated metabolites and cell types in the forestomach. (A to C) Principal coordinates analysis (PCoA) profile of epithelial bacterial diversity between adult (AD) and newborn (NB) groups in the rumen (A), reticulum (B), and omasum (C). (D) The Venn diagram represents the overlaps of the metabolites that have high co-occurrence probabilities with *Megasphaera* among the rumen, reticulum, and omasum. (E) The co-occurrence probabilities of the 3 metabolites in the rumen, reticulum, and omasum. (F) Gene scoring analysis of Meta-MC between NB and AD groups using the methyltransferase complex gene set in the rumen, reticulum, and omasum.

In addition to the regulation of the host itself, early development of the rumen was also triggered by commensal microbes [7]. A recent study has suggested that during early life development, the host epigenetic modification (DNA methylation) by symbiotic microbiota represents a fundamental and broad level of regulation [22]. As outlined above, we found that the MC transcriptional plasticity was associated with the activity of methyltransferase complex in the forestomach tissues of NB cattle. Therefore, we next aimed at exploring mucosal microbiota involved in DNA methylation of host cells. Firstly, we accomplished 16S ribosomal RNA (rRNA) gene sequencing to analyze the mucosal bacteria in the forestomach tissues at NB and AD stages. We found that the mucosal bacterial profiles of NB and AD were distantly clustered by a principal coordinates analysis plot from the rumen, reticulum, and omasum tissues (Fig. 6A to C). At the genus level, we found that 5, 5, and 8 bacteria genera showed higher relative abundances in the rumen, reticulum, and omasum of NB calves, respectively (Fig. S7), of which 3 (*Megasphaera*, *Streptococcus*, and *Enterococcus*) were shared by the rumen, reticulum, and omasum of NB calves.

*Streptococcus* includes facultatively anaerobic bacteria that could participate in the creation of a reduced environment for anaerobic microbiota [23,24], which might facilitate early colonization of anaerobic microbiota in the forestomach tissues of calves after birth. Emerging evidence has suggested that *Megasphaera* was associated with the development of calf rumen epithelium [7,8]. Thus, we next performed a metabolomics analysis in NB and AD forestomach mucosal tissues (see Materials and Methods), and identified 718, 722, and 722 metabolites in the rumen, reticulum, and omasum, respectively (Tables S4 and S5). Then, we used a microbe-metabolite vectors (mmvec) neural network analysis [25] to identify the *Megasphaera*–host interaction by calculating the co-occurrence probabilities between the *Megasphaera* genus and metabolites in the forestomach mucosal tissues (see Materials and Methods). We found that 136, 129, and 138 metabolites had high co-occurrence probabilities (the inferred conditional probabilities > 1) with the *Megasphaera* genus in the rumen, reticulum, and omasum, respectively (Table S6). Only 5 metabolites, including the dimethyl fumarate, carnitine C8:0, propionylcarnitine, N-Alpha-acetyllysine, and 5-methylcytosine, showed high co-occurrence probabilities with the *Megasphaera* genus and were shared in the rumen, reticulum, and omasum (Fig. 6D and E). Three of them (propionylcarnitine, N-Alpha-acetyllysine, and 5-methylcytosine) are microbiota-dependent metabolites according to a reference library of microbiota-dependent metabolites [26]. Finally, we performed a gene set score analysis to calculate the molecular signature scores of methyltransferase complex gene sets of MCs from NB and AD in the forestomach tissues. The results showed that MCs in the NB group had high methyltransferase complex scores compared with the AD group in the forestomach tissues (Fig. 6F). Thus, the *Megasphaera* genus may regulate the transcriptional plasticity of forestomach epithelial cells by epigenetic regulation in NB calves. Further studies to reveal the underlying molecular mechanisms are required.

### Age- and species-specific novel cell type enables cell stemness maintenance in NB cattle

**Fig. 7.**
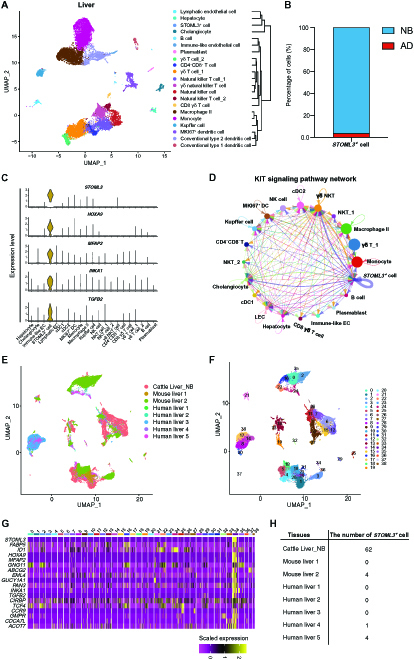
The *STOML3^+^* cell enables cell stemness maintenance in the newborn (NB) liver. (A) Uniform manifold approximation and projection (UMAP) maps of the liver tissues and the results of hierarchical cluster analysis of cell types on the right panel. (B) The bar graph shows that the *STOML3^+^* cell is specifically present in the NB liver. (C) The violin plot shows the representative marker genes of the *STOML3^+^* cell. (D) The inferred KIT signaling networks in the NB liver single-cell dataset. The circle sizes and edge width represent proportional to the number of cells in each cell group and the communication probability, respectively. (E and F) UMAP plot of liver cells from different species, colored by species tissues (E) and cell clusters (F). (G) The heatmap shows the representative marker genes used to identify the *STOML3^+^* cell among the cell clusters. (H) The numbers of the *STOML3^+^* cell in the cattle, human, and mouse liver datasets. DC, dendritic cell; cDC1, conventional type 1 dendritic cell; cDC2, conventional type 2 dendritic cell; NK, natural killer; NKT, natural killer T; LEC, lymphatic endothelial cell.

In addition to the forestomach tissues, the function of the liver also undergoes dramatic changes during postnatal functional maturation in ruminants. The physiological changes in the liver of NB and AD cattle could be observed and appreciated obviously [4]; however, the driving factors remain poorly understood. After analyzing liver tissues from NB and AD, we found a previously undescribed cell type, the *STOML3^+^* cell, in the liver of NB cattle (Fig. 7A and B). When the similarities between liver cell types were compared using the hierarchical cluster analysis, the *STOML3^+^* cell clustered much closer to cholangiocytes, hepatocytes, and lymphatic endothelial cells than to other cell types (Fig. 7A). This cell type has many highly expressed marker genes, such as *STOML3*, *HOXA9*, *MFAP2*, *INKA1*, and *TGFB2* (Fig. 7C), suggesting that it may have a cell stemness maintenance function. For example, *HOXA9* is involved in regulating cell stemness [27]. Similarly, *INKA1* is a promoter of stem cell latency [28]. By constructing ligand–receptor interaction maps using the CellChat [29], we found that the *STOML3^+^* cell predominantly interacts with itself and a cholangiocyte through KIT signaling (Fig. 7D). KIT signaling has been uncovered to be involved in the differentiation, proliferation, and migration of many cell types and thereby plays an important role in stem cell maintenance [30]. Collectively, this novel cell type found in NB liver tissue may contribute to the maintenance of cell stemness in the hepatic microenvironment.

Since NB calves are considered functionally monogastric animals and their liver tissues have many functions that were similar to that in monogastric species [4,31], thus, we next explored by cross-species comparison based on single-cell transcriptomics whether the *STOML3^+^* cell also exists in human and mouse liver tissues (see Materials and Methods). We integrated 5 human [32] and 2 mouse liver single-cell datasets [33] with our NB cattle liver dataset and then performed Seurat clustering analysis. The UMAP projection of cells showed a certain extent of cell population variance across cattle, human, and mouse liver tissues (Fig. 7E). In total, we identified 39 distinct clusters (Fig. 7F), and cluster 34 was identified as the *STOML3^+^* cell on the basis of highly expressed marker genes (Fig. 7G). Interestingly, the *STOML3^+^* cell was mainly observed in NB cattle liver tissue and was almost absent in human and mouse liver tissues (Fig. 7H). Although 4 and 5 cells belonging to the *STOML3*^+^ cell cluster were observed in the mouse and human liver tissues, respectively, however, they were regarded as mislabeled cells because of the unexpressing marker genes *STOML3*, *MFAP2*, *INKA1*, and, *TGFB2*. This is a predictable artifact of the annotation scheme because entire clusters instead of individual cells were annotated in each tissue. Sufficiently small populations of cells that were algorithmically grouped with a more populous cell type will be misannotated [34]. These results indicate that the *STOML3^+^* cell did not exist in human and mouse liver tissues and could be NB calves-specific. Further deeper understanding of the *STOML3^+^* cells is required in the future.

## Discussion

Constructing age-dependent cell atlases for 13 different cattle tissues provided a comprehensive understanding of cell compositions and their dynamic changes. We observed the landmarks of single-cell gene expression in different bovine tissues to systematically annotate the cattle cell types and established the CCL website (http://cattlecelllandscape.zju.edu.cn) for free access to substantial cell-type-specific gene markers, which may become a valuable reference source in the effective annotation of cell types for the broad research community.

In the life of ruminants, one of the most important physiological transformations is from the preruminating to the ruminating stage. Several studies have attempted to identify the underlying molecular mechanisms mediating postnatal maturation of ruminating calves using bulk RNA sequencing [7,35]; however, these studies only focused on the rumen and offered some opposite findings. As an example, Connor et al. [35] found that the *PPARA* was a vital gene for the rumen epithelial development during the transition from preruminating to ruminating, but in the study of Malmuthuge et al. [7], the *PPARA* did not exhibit a pattern of temporal expression with calf age. Those differences may be attributed to cell heterogeneity at the transcriptomic level. Moreover, the complicated biological process from preruminating to ruminating states is likely orchestrated by several tissues. In addition to the rumen, the reticulum, omasum, abomasum, small and large intestine tissues, and other metabolic tissues undergo wide transformation on the gene expression and physiological functions during this process [4,5,18]. In the current study involving the cell stemness of epithelial cells across 13 cattle tissue types, we observed that the abomasum, duodenum, cecum, colon, and rectum had lower entropy (low cell stemness) in the NB stage compared with the AD stage. The lower entropy represents a skewed distribution of expressed genes in functional gene clusters of individual cells that are associated with well-defined cell fates and functionalities [11]. As such, the abomasum and intestinal tissues (duodenum, cecum, colon, and rectum) may undergo rapid functional specificity after birth to be physiologically adapted to colostrum and milk.

Contrary to the results in the abomasum and intestinal tissues, our results showed that in the early postnatal period, higher levels of cell stemness were measured in the forestomach (rumen, reticulum, and omasum) epithelial cells. Organogenesis of complex organisms is dependent on stemness states of individual cells [10,11]. The high level of cell stemness of the forestomach epithelial cells at the NB stage may be necessary for the marked morphological and functional changes of these organs after birth. As far as we know, no studies have reported the difference in stemness states between epithelial cells of NB and AD cattle because traditionally the cellular stemness status is measured on the basis of the expression level of known differentiation markers [10,11] or by the cellular morphological features [36], in which the unique cellular differentiation markers of bovine cell lineages remain underinvestigated [12,13]. On the basis of the newly acquired single-cell data, we uncovered that MCs (epithelial progenitor-like cells) in the forestomach tissues (rumen, reticulum, and omasum) had the highest stemness and showed more diverse differentiation patterns in the NB stage, indicating that the rapid tissue development in the early postnatal life of calves was cell-type-driven. The higher activities of the epigenetic modification of these cells suggest that the epigenetic status is involved in the process of forestomach postnatal development because each cell has its differentiation patterns that are influenced by its epigenetic status [37]. Taken together, our results offer an age-dependent multitissue representation paradigm of cell stemness plasticity, which is associated with the major functional conversion from the abomasum and gut to the forestomach that is considered to be the basis of maturation of the ruminating system. Further analyses are required to verify the proposed impacts of cell stemness plasticity observed in our current study and the exact molecular mechanisms.

By integrating microbial metagenomics and host transcriptomics, a previous study revealed that parts of the host mRNA transcripts were responsive to commensal microbes during the early development of the rumen [7]. In the current study, by integrating epithelial microbiome, metabolome, and single-cell transcriptome, we found that the involvement of the mucosal *Megasphaera* genus in the proliferation and development of the epithelium may be related to propionylcarnitine, N-Alpha-acetyllysine, and 5-methylcytosine in the rumen, reticulum, and omasum tissues of NB calves. The synthesis of propionylcarnitine requires the participation of lysine and its derivatives, and the propionylcarnitine improves endothelial cell function, in enhancing the blood flow of the blood vessels [38], thereby supporting nutrient supply and utilization. This may closely relate to calf forestomach epithelial tissue growth because the nutrient supply of cells in the forestomach tissues that have not yet matured up to rumination function depends on nutrient transport in blood vessels. DNA methylation plays a vital physiological role in the regulation of gene expression of cells [39]. The *Megasphaera* genus had a high co-occurrence probability with 5-methylcytosine, and epithelial progenitor-like cells had higher activity of the methyltransferase complex, implying that the *Megasphaera* genus may participate in regulation of differentiation and proliferation of epithelial progenitor-like cells in the NB rumen, reticulum, and omasum tissues by epigenetic regulation. In addition, the *Megasphaera* can convert lactate into butyrate (1 kind of short-chain fatty acid) that is responsible for modulating the ten-eleven translocation methylcytosine dioxygenase enzymatic activity that are involved in DNA methylation by affecting TCA intermediates [22,40]. However, more studies are required to unravel the underlying molecular mechanism.

In the time of the early life stage of calves, the GIT is continually communicating with other organs, such as liver [18]. Although the liver of NB ruminants has many functions that were similar to that of monogastric species [31], we found an NB cattle-specific cell type, the *STOML3^+^* cell, which plays a crucial role in stemness maintenance of itself and cholangiocytes. Cholangiocytes are highly dynamic epithelial cells, and their functional physiological modification of hepatocyte-derived bile is a functional response of the liver to diets [41]. As such, cross-talks between the *STOML3^+^* cell and the cholangiocyte may contribute to accommodating digestive adaptations during the transition period. Why *STOML3^+^* cell only exists in cattle liver in the early postnatal period and whether it affects the liver maturation process in response to GIT development warrant further investigations.

There are certain limitations in our current study, which need additional experimental work and new tools. Although a previous study reported that rumen epithelial tissues of calves before and after weaning and corresponding cell clusters were generally similar [42], more tissue-type samples from different time points should be collected and analyzed for an even better understanding of the process. The comprehensive marker genes of the *STOML3^+^* cell were developed in our study. However, we failed to verify the *STOML3^+^* cell by performing the immunohistochemistry experiments using polyclonal antibodies against humans since useful immunohistochemistry reagents (polyclonal or monoclonal antibodies) with specificity to bovine cells are absent, but we believe that this issue will be solved in near future.

Taken together, we delineated a comprehensive single-cell compendium covering 13 tissue types of NB and AD cattle. We have identified cell heterogeneity for many tissues that have not been well characterized yet and constructed the CCL website to explore in detail the results of single-cell datasets in different bovine tissue types. Moreover, the alterations in cell type composition, cell-cell communication, and cell stemness on multiple temporal and spatial scales were observed, collectively providing novel and fundamental insights into postnatal ruminating maturity.

## Materials and Methods

### Experimental animals

All the experimental procedures were approved by the Animal Care Committee at Zhejiang University (Hangzhou, China). scRNA-seq datasets from AD rumen, reticulum, omasum, abomasum, ileum, rectum, liver, salivary gland, and mammary gland were obtained from our previous study [17]. The AD duodenum, jejunum, cecum, and colon were collected from a single lactating Holstein dairy cow. Three NB calves of similar weight were humanely euthanized in a surgery room to collect the tissue samples. Clinical information on all the animals is available in Table S1.

### Single-cell suspension preparation and RNA sequencing

The AD duodenum, jejunum, cecum, and colon samples and the NB reticulum, omasum, abomasum, duodenum, jejunum, ileum, cecum, colon, rectum, liver, and salivary gland tissue samples were freshly collected from the cattle that were humanely euthanized.

After removal of the outer muscle layers, the AD duodenum, jejunum, cecum, and colon tissue samples were cut into 1-mm pieces and treated with different enzymes for different durations: the duodenum and cecum samples were dissociated using the Lamina Propria Dissociation Kit (Miltenyi Biotec) for 30 min at 37 °C; the jejunum and colon samples were dissociated using the Multi Tissue Dissociation Kit 2 (Miltenyi Biotec) for 41 min at 37 °C. Then, 10% of fetal bovine serum (FBS) was added to stop the digestion, followed by a step of filtration with the 40-μm sieve. Next, after centrifugation at 300 × g for 5 min at 4 °C and after washing with 1× Dulbecco’s phosphate-buffered saline (DPBS) containing 2% FBS and 0.2 mM EDTA, the samples were treated with Red Blood Cell Lysis Solution (Miltenyi Biotech) for 2 min. Dissociated cells were washed with 1× DPBS containing 2% FBS and 0.2 mM EDTA, centrifuged at 300 × g for 5 min at 4 °C, and resuspended in 1× DPBS containing 10% FBS and 0.2 mM EDTA.

To prepare the single-cell suspensions of the NB reticulum, omasum, abomasum, small intestines (duodenum, jejunum, and ileum), and large intestines (cecum, colon, and rectum) tissue samples, we referred to the protocols of our previous study [17]. Specifically, we first removed the muscle layers of samples and then chopped the samples into 10 × 0.5 mm^2^ pieces. After incubating with 20 mM EDTA for 30 min at 37 °C, samples were rinsed with DPBS and minced into 1-mm pieces. Tissue samples were incubated in a 37 °C water bath for 5 min with 0.25% Trypsin-EDTA (Gibco) and then placed in ice for 2 min, while the digestion was stopped by adding the prechilled Hank’s balanced salt solution (HBSS). Samples were centrifuged at 4 °C for 2 min with 300 × g and removed the supernatant and then washed twice with cold HBSS. Next, for the NB reticulum, omasum, and abomasum samples, the multiple enzymes (1.5 mg/ml collagenase IV, 1.5 mg/ml collagenase I, 100 U/ml hyaluronidase, 50 U/ml deoxyribonuclease I [DNase I], and 1.5 mg/ml dispase) were added and treated for 30 min at 37 °C. For the NB small intestines (duodenum, jejunum, and ileum) and large intestines (cecum, colon, and rectum) tissue samples, the multiple enzymes from the Propria Dissociation Kit (Miltenyi Biotec) were added and treated for 30 min at 37 °C. The digestion was terminated by adding 10% FBS, followed by a step of filtration with the 70- and 30-μm SmartStrainer (Miltenyi Biotec). After centrifugation at 300 × g for 5 min at 4 °C, samples were resuspended in 2 ml of HBSS. After centrifugation at 4 °C for 5 min with 300 × g and washing twice with 1× phosphate-buffered saline (PBS) with 0.04% bovine serum albumin (BSA). Finally, samples were centrifuged at 4 °C for 5 min with 300 × g and resuspended in 1× PBS with 0.04% BSA.

Single-cell suspensions of the liver and salivary gland tissues of NB calves were prepared according to the protocol of our previous study [17]. For the NB liver tissue, the sample was transferred into cold DPBS, chopped into 1-mm pieces, and treated with 1.5 mg/ml collagenase I, 20 U/ml papain, and 50 U/ml DNase I for 20 min at 37 °C. The dissociated cells were then passed through 70- and 30-μm SmartStrainer, centrifuged at 4 °C for 5 min with 300 × g, and resuspended with 2 ml of cold HBSS. Dissociated cells were centrifuged for 5 min at 300 × g, washed twice with 1× PBS with 0.04% BSA at 4 °C, and then resuspended in 1× PBS with 0.04% BSA. For the NB salivary gland tissue, the sample was isolated from the parotid gland was placed into cold 1× PBS, and then the blood and fat was removed. The sample was cut into 0.5-mm pieces and incubated with 1.5 mg/ml collagenase II, 1.5 mg/ml dispase, and 50 U/ml DNase I at 37 °C for 20 min. Dissociated cells were passed through 70- and 30-μm SmartStrainers. After centrifugation at 4 °C for 5 min with 300 × g, dissociated cells were resuspended with 300 μl of RPMI 1640 medium (Gibco) and treated with 3 ml of Red Blood Cell Lysis Solution for 3 min. Then, dissociated cells were centrifuged at 4 °C for 5 min with 300 × g and resuspended with 2 ml of RPMI 1640 medium. After centrifugation for 5 min with 300 × g, dissociated cells were washed twice with 1× PBS with 0.04% BSA at 4 °C and then resuspended with 1× PBS with 0.04% BSA.

The Countess II Automated Cell Counter was used for the assessment of the viability of single-cell suspension via trypan blue. If the cell viability was low, then the MACS Dead Cell Remove Kit (Miltenyi Biotec) was used to remove dead cells according to the manufacturer’s recommendations. Dissociated live cells were captured to construct the libraries using the Chromium Single Cell 3' Reagent Kits v3 (10x Genomics) following the manufacturer’s recommendations. The Agilent Bioanalyzer High Sensitivity chip was used to check the quality of the libraries, and then libraries were sequenced on the NovaSeq 6000 platform (150-bp pair-ended manner).

### scRNA-seq data analysis

We generated 15 new scRNA-seq datasets in the present study. The CellRanger (version 3.1.0) was used for sample demultiplexing, barcode processing, and single-cell 3’ gene counting. The reads of scRNA-seq data were aligned to the ARS-UCD1.2 cattle reference genome. Single-cell transcriptomics datasets, generated using 10x Genomics, of AD rumen, reticulum, omasum, abomasum, ileum, rectum, liver, salivary gland, and mammary gland as well as the NB rumen and mammary gland were collected from our a previous study [17] and in-house lab datasets. A total of 30 scRNA-seq datasets representing multiple tissues were analyzed individually. For each dataset, high-quality cells with 500 to 4,000 genes detected, unique molecular identifier counting < 50,000, and the ratio of mitochondrial gene < 40% were retained using the Seurat (version 4.0.3) R package [43] for further analysis. The doublets in each dataset were also removed using the DoubletFinder package (version 2.0.3) [44].

To create the cross-tissue temporal cell atlas, the aforementioned filtered, nonscaled Seurat objects of NB and AD cattle datasets were imported and merged into a Seurat object. The merged object was normalized (NormalizeData function of Seurat) and scaled to regress out the cell cycle signature (computed using Seurat’s CellCycleScoring function and scaled using Seurat’s ScaleData, vars.to.regress = c("S.Score", "G2M.Score")). The principal component analysis was performed (RunPCA function of Seurat), and next, the batch effect was corrected using the Harmony R package (version 0.1.0) [45]. The FindClusters function of Seurat with an appropriate resolution was used to accomplish cell clustering. The UMAP embedding algorithm (RunUMAP function) or t-distributed stochastic neighbor embedding algorithm was used to visualize cells. Finally, the FindAllMarkers function of Seurat was performed to identify marker genes (|"avg_logFC"| > 0.25 and "p_val_adj" < 0.05).

To further investigate the dynamics between the NB and AD groups, we merged the nonscaled Seurat objects of the NB and AD datasets for each tissue type and performed cluster analysis separately following the same analysis pipeline (but not regressing out the cell cycle signature) to generate age-dependent cell atlases for each tissue type. We further manually removed cell clusters (potential doublets) that had overlapping gene profiles of multiple different cell types and excluded the hemocytes. The rumen age-dependent cell atlas was collected from our previous study [21].

The single-cell entropy (scEntropy) of individual epithelial cells in NB and AD cattle tissue was quantitatively measured on the basis of scRNA-seq datasets using the SLICE pipeline [11] with some modifications. In brief, using Kappa statistics [46], we calculated the functional similarity of genes in the *Bos taurus* GOTERM_BP_FAT subset that was collected from the DAVID Functional Annotation dataset [47] to generate the genome-wide gene-to-gene functional similarity matrix of *Bos taurus*. Next, we replaced the functional similarity matrix of human with that of *Bos taurus*, and other analysis steps were performed with default parameters following the SLICE pipeline. The differences between NB and AD cattle were assessed using the analysis of variance, with a *P* value < 0.0001 as the threshold for significance. *, **, and *** represent *P* < 0.0001, *P* < 1.0 × 10^−100^, and *P* < 1.0 × 10^−200^, respectively.

To assess the similarities of epithelial cell subtypes in the forestomach tissues, the expression matrices (raw counts) of epithelial cell subtypes of the rumen, reticulum, and omasum at NB and AD cattle were merged, respectively. The 2 merged expression matrices were separately used to perform the MetaNeighbour analysis [20] with default parameters. The epithelial cell subtypes were considered to be conserved if AUROC scores between them were higher than 0.95.

We used the Monocle3 [48] to model differentiation trajectories of reticulum epithelial cell types in NB and AD cattle with default parameters following the general pipeline (https://cole-trapnell-lab.github.io/monocle3/). The CellChat [29] with default parameters was used to perform ligand–receptor interaction analysis to recognize the inferred cellular communication patterns in the NB liver scRNA-seq data. The “AddModuleScore” function of Seurat (version 4.0.3) was used to perform gene set scoring analysis to calculate the signature scores on the gene sets in the MCs between NB and AD cattle in the forestomach tissues. The 2-sided Wilcoxon rank sum test was used to evaluate the differences in the signature scores between NB and AD cattle. Detailed information of the gene sets “DNA repair” and “methyltransferase complex” are listed in Table S7.

To explore the *STOML3^+^* cell across different species, we downloaded the dataset from 5 human liver single-cell datasets [32] (https://github.com/BaderLab/HumanLiver) and 2 mouse liver single-cell datasets [33] (GSM3714747 and GSM3714748 in the National Center for Biotechnology Information Gene Expression Omnibus accession GSE129516). We firstly performed expression matrix preprocessing separately for the 3 species using the Seurat (version 4.0.3) and retained the detected homologous genes of these 3 species in each scRNA-seq dataset. Next, we merged the datasets into a Seurat object and performed cell clustering analysis to come up with 39 cell clusters.

### CCL website construction

The main CCL website was completed by PHP language and MySQL. The CCL website offers 2 function modules: the “Landscape Datasets” module and the “Marker Genes Search” module. The “Landscape Datasets” module enabled the visualizations for cell types of 13 different tissue types and allowed investigation the cell-type populations’ heterogeneity from NB and AD cattle. The “Marker Genes Search” module provided the top 100 highly expressed genes of each cell type across various tissues and also displayed the expression of marker genes presented as a heatmap at the cell-type averaged level. More details about the CCL website are available at http://cattlecelllandscape.zju.edu.cn.

### Differential expression analysis

The Wilcoxon rank-sum test as implemented in the Seurat’s “FindMarkers” function [43] was performed to identify DEGs of MC between NB and AD cattle. Only genes with |LogFC| > 0.5 and adjusted *P* value < 0.05 were considered as DEGs.

### Enrichment analysis

The gene sets that are enriched in MCs of the reticulum and omasum were identified by the GSEA using the “GSEA” function of clusterProfiler R package [49]. The “enrichGO” function of clusterProfiler R package was applied to the performance of Gene Ontology (GO) term enrichment analysis based on the dataset “org.Bt.eg.db”.

### Random forest classifier

The ClassifyCells function that is implemented in the Seurat R Package (version 2.2.0) was used to perform the random forest classification with default parameters. We grouped the subtypes of MC identified from the rumen samples as the training class, which was subsequently applied to the MC from the reticulum and omasum samples.

### 16S rRNA gene sequencing and analysis

Six animals (3 per group) were selected to collect the mucosa. The mucosal bacterial profiles of the rumen in the NB and AD cattle were collected from our previous study [21]. Total DNA of the mucosal microbiota was extracted from each reticulum and omasum epithelial tissue using the E.Z.N.A.® DNA Kit (Omega Bio-Tek) according to the manufacturer’s instructions. The primer pairs 338F (5′-ACTCCTACGGGAGGCAGCAG-3′) and 806R (5′-GGACTACHVGGGTWTCTAAT-3′) were used to amplify the hypervariable region (V3-V4) of the bacterial 16S rRNA gene. After polymerase chain reaction amplification, all amplicon libraries were sequenced on an Illumina MiSeq platform using the paired-end 2 × 300-bp protocol. The paired-end reads were merged using the FLASH (version 1.2.11) [50]. The data were processed using the QIIME2 software package [51]. The DADA2 plugin [52] was used to perform further quality control and chimera removal and to produce the amplicon sequence variant (ASV) feature tables. The representative sequences of these ASVs were identified and assigned to the bacteria database of SILVA (Release138, http://www.arb-silva.de) and clustered at 99%. We performed linear discriminant analysis effect size based on the ASVs that were merged to the genus level to identify bacterial taxa that were significantly (linear discriminant analysis > 3.5, *P* < 0.05) enriched in NB or AD cattle of the reticulum and omasum.

To predict the relationship between the genus *Megasphaera* and metabolites in the rumen, reticulum, and omasum tissues, the microbe–metabolite vectors (mmvec) neural networks analysis was performed [25]; the analysis pipeline using default parameters was performed to determine the conditional probability that each metabolite was present given the presence of genus *Megasphaera* and based on the microbial sequence counts and the metabolite relative concentration.

### Metabolomics analysis

The same animals were selected to conduct metabolomics analysis. The mucosal metabolite profiles of the rumen in the NB and AD cattle were collected from our previous study [21]. The epithelial tissues of the reticulum and omasum were homogenized with 1,000 μl of 70% (v/v) ice-cold methanol/water and cold steel balls at 30 Hz for 3 min and then were whirled for 1 min without steel balls. After resting for 15 min and centrifugation at 4 °C, 12,000 rpm for 10 min, the supernatant of the sample was collected for liquid chromatography-tandem mass spectrometry analysis. We performed the electrospray ionization quadruple trap-tandem mass spectrometry analysis for the supernatant of each sample. The operation parameters of the electrospray ionization source were as follows: ion spray voltage, 5,500 V (positive) and −4,500 V (negative); source temperature, 500 °C; ion source, gas I and gas II; curtain gas were set at 50, 50, and 25 psi, respectively; ion spray voltage, 5,500 V (positive) and −4,500 V (negative); the collision gas was set to high. The instrument tuning and mass calibration were accomplished with 10 and 100 μmol/l polypropylene glycol solutions in QQQ and LIT modes, respectively. The Analyst software (version 1.6.3) was used to analyze the mass spectrometric data.

## Data Availability

The raw data of scRNA-seq of the reticulum, omasum, abomasum, duodenum, jejunum, ileum, cecum, colon, rectum, liver, and salivary gland samples from NB calves and those of duodenum, jejunum, cecum, and colon samples from AD cows have been deposited to the Gene Expression Omnibus database (accession number GSE192807), and the secure token “mjejmiqabnifvsn” allows review of record GSE192807. The raw data of scRNA-seq of rumen tissues was collected from GSE183285, and the secure token “mdotewyuldqzbmx” allows review of record GSE183285. The raw data of the NB mammary gland was collected from our previously published data in the Sequence Read Archive database under accession number SRX9140855. The raw data of the 16S rRNA gene sequencing in the current study have been deposited to the BioProject PRJNA846365 (https://www.ncbi.nlm.nih.gov/bioproject/ PRJNA846365/). The processed data are also available for interactive viewing on the CCL website, available at http://cattlecelllandscape.zju.edu.cn. This study did not generate any unique code.
