# Supplementary material for: Age- and Microbiota-Dependent Cell Stemness Plasticity Revealed by Cattle Cell Landscape
Source: Research (Wash D C). 2023 Jan 13;6:0025. doi: 10.34133/research.0025 (PMC10076005; doi:10.34133/research.0025)
Supplement: Supplementary Materials — Supplementary 1. Figs. S1 to S7 Supplementary 2. Tables S1 to S7 [file research.0025.f1.docx]

**
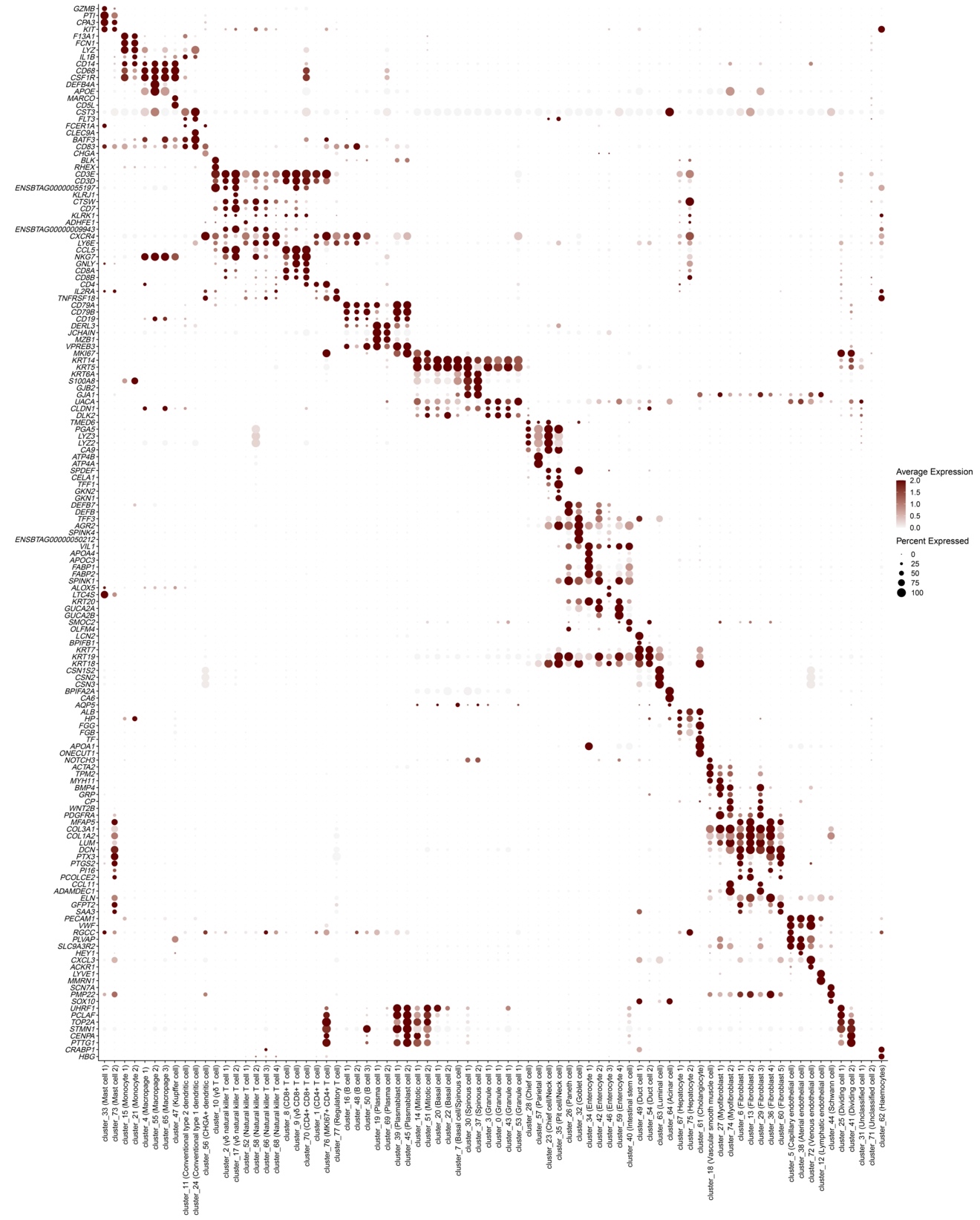
**

**Figure S1. Dot plots showing the expression of representative marker genes for each cell type.**

**
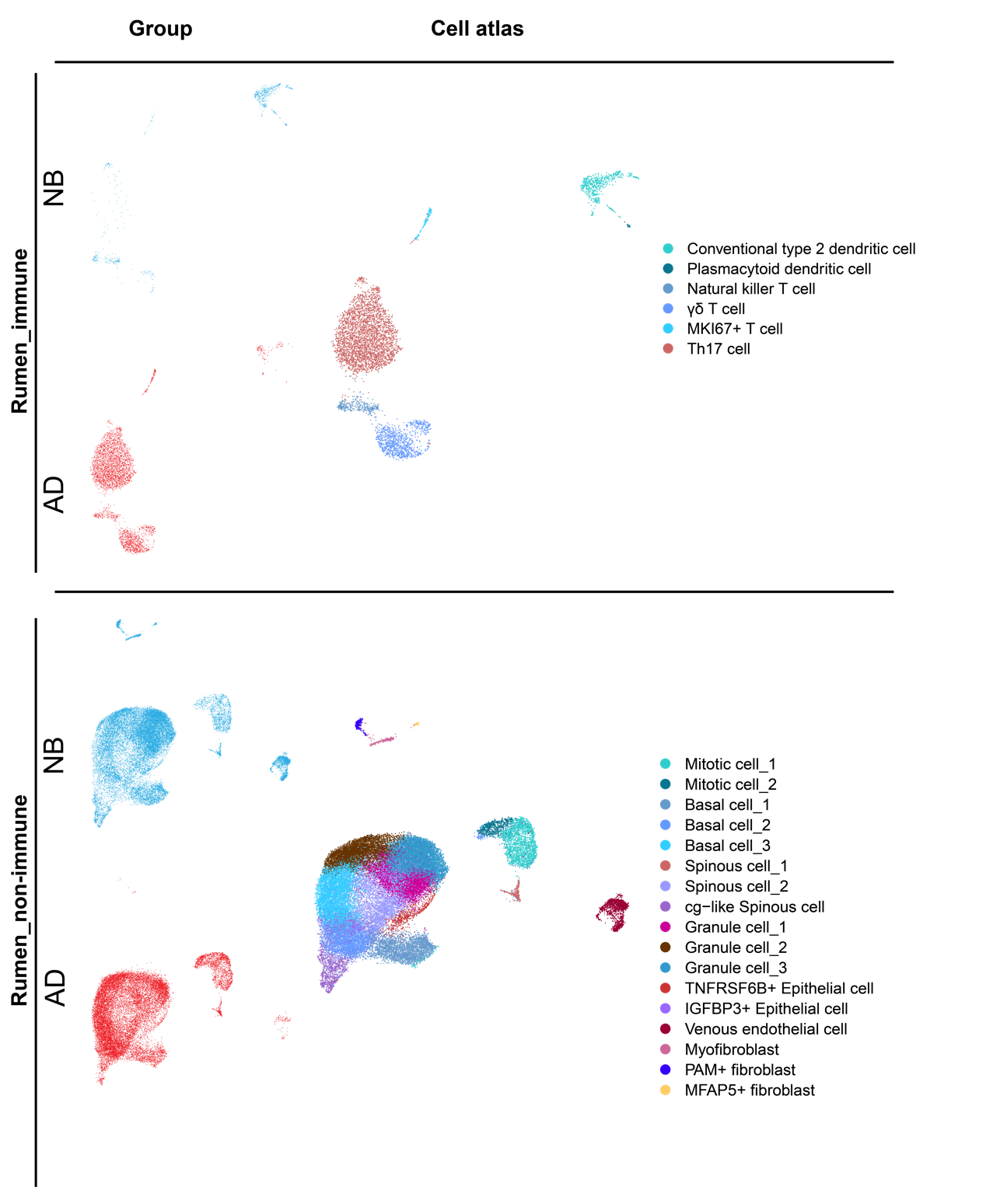
**

**Figure S2. Cell atlases of all cell types in the rumen tissue among the two groups (NB, blue; AD, red). The rumen immune- and non-immune- cell atlases were collected from our previous study [21].** NB: newborn; AD: adult.


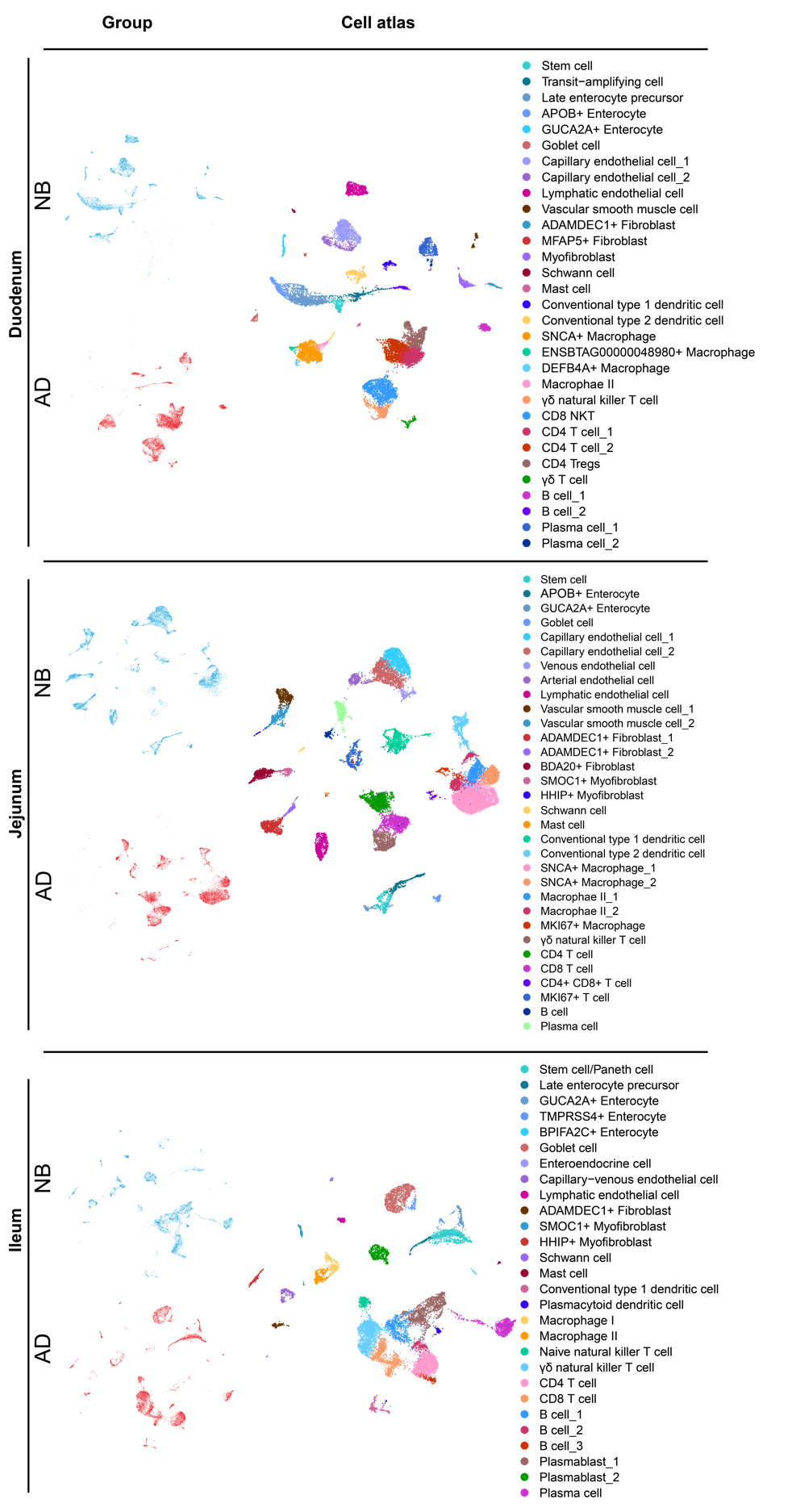


**Figure S3. Cell atlases of all cell types in the duodenum, jejunum, and ileum tissues among the two groups (NB, blue; AD, red).** NB: newborn; AD: adult.


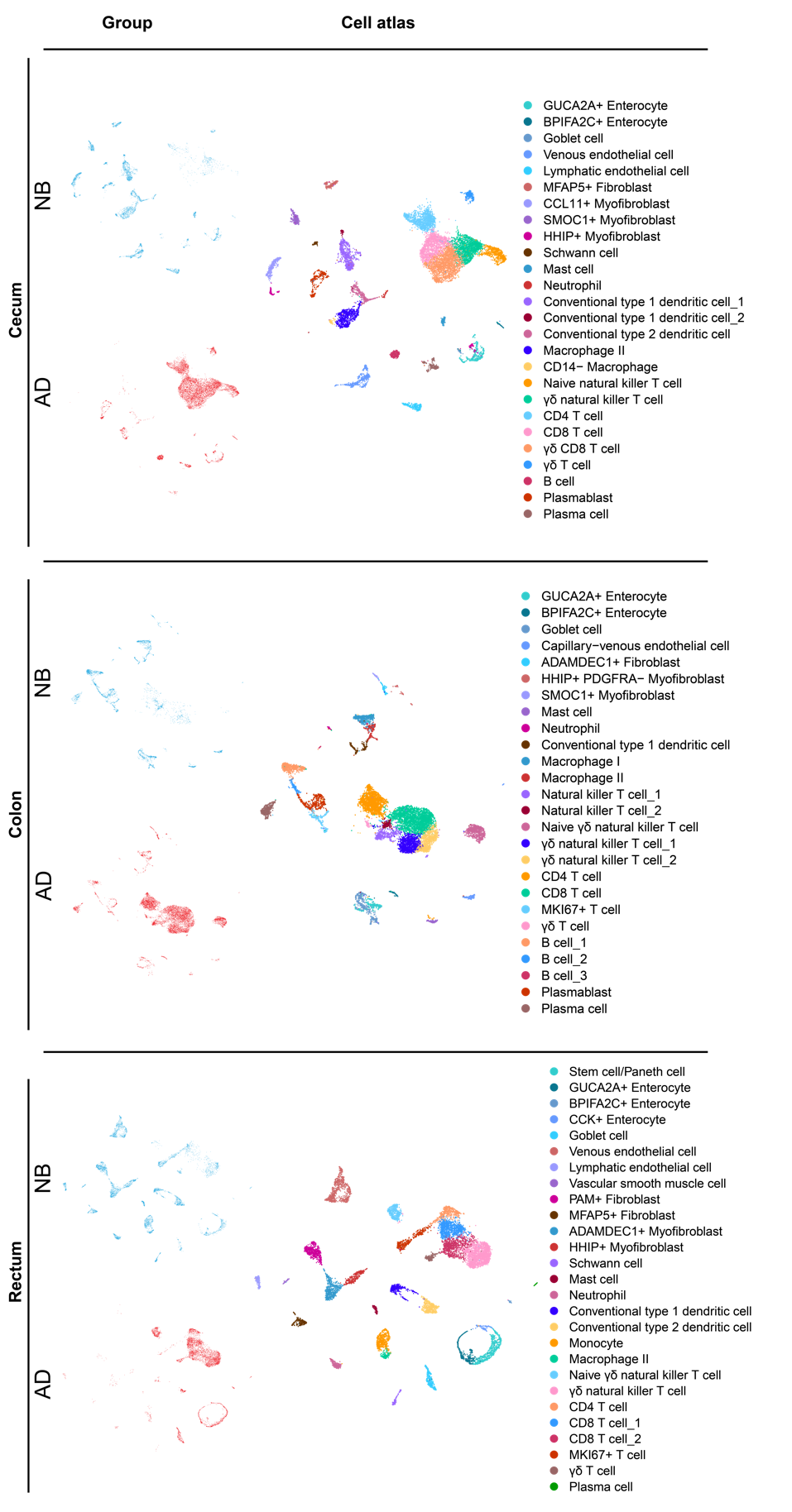


**Figure S4. Cell atlases of all cell types in the cecum, colon, and rectum tissues among the two groups (NB, blue; AD, red).** NB: newborn; AD: adult.

**
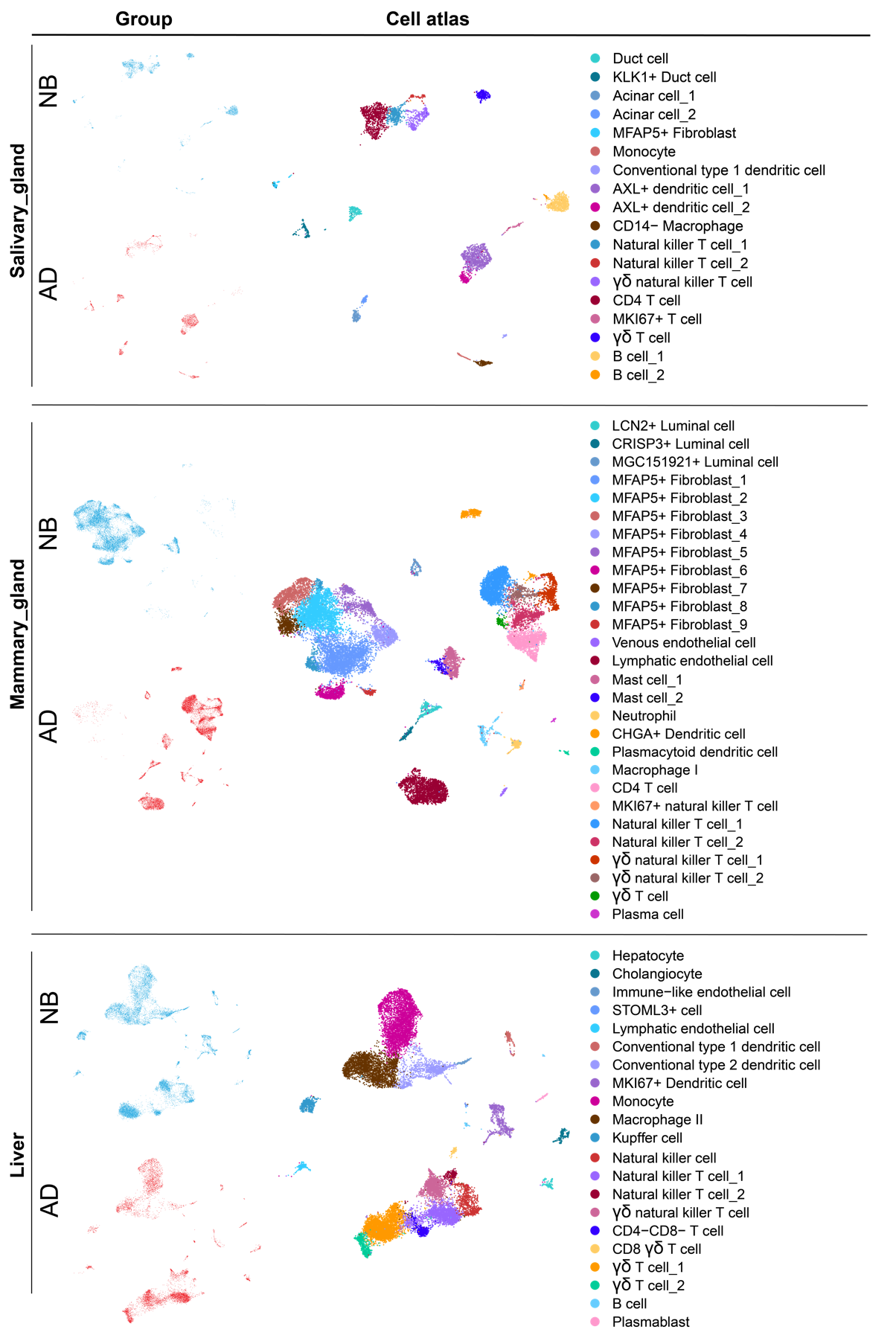
**

**Figure S5. Cell atlases of all cell types in the salivary gland, mammary gland, and liver tissues among the two groups (NB, blue; AD, red).** NB: newborn; AD: adult.

**
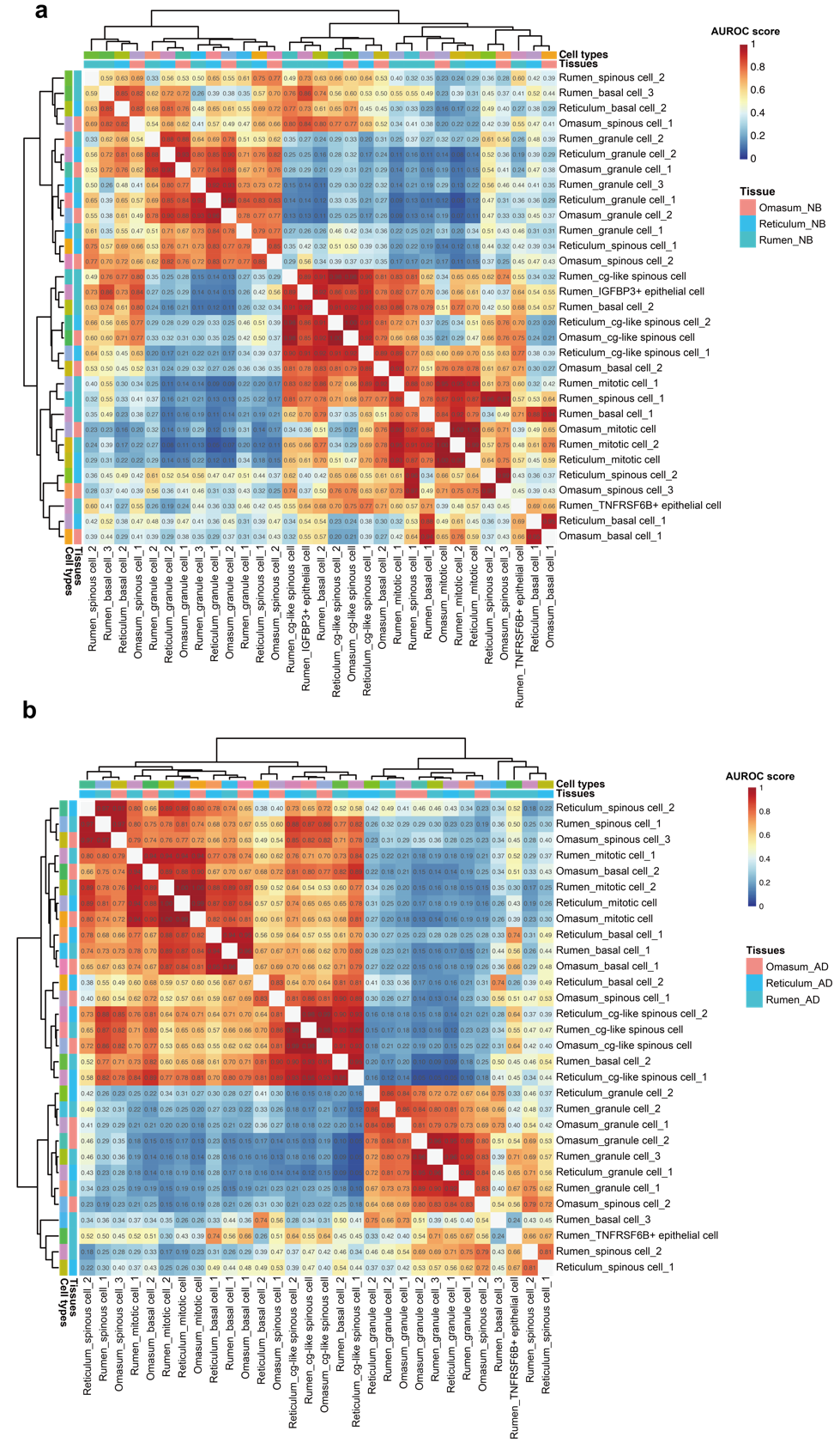
**

**Figure S6.** Correlation of gene expression among the rumen, reticulum, and omasum epithelial cell types at NB (**a**) and AD (**b**) stages. AUROC scores were used to measure the similarity of cell types: red, high correlation; blue and yellow, low correlation. Note that the diagonal value has no practical significance according to the scoring system of the algorithm. For the purpose of visualization, it is filled with light gray. AUROC: area under the receiver operating characteristics; NB: newborn; AD: adult.


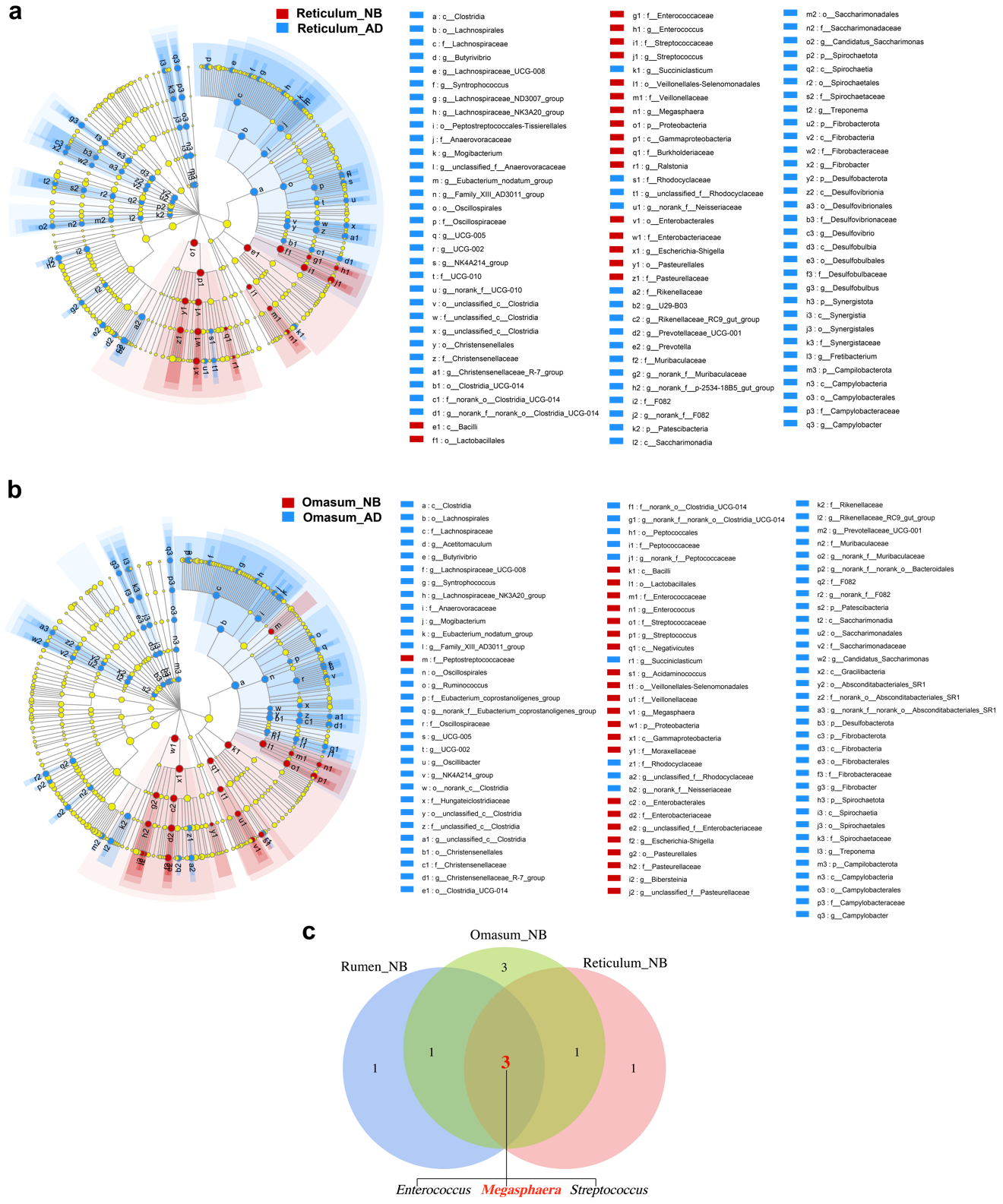


**Figure S7.** Bacterial taxa differed significantly different between NB and AD groups in the reticulum (**a**) and omasum (**b**) tissues. (**c**) The Venn diagram represents the overlaps of epithelial bacterial genera that had a higher relative abundance in the NB groups among the rumen, reticulum, and omasum. The different bacterial taxa identified between NB and AD groups in the rumen were collected from our previous study [21]. NB: newborn; AD: adult.
